# Parallel transmission for ultrahigh‐field imaging

**DOI:** 10.1002/nbm.3313

**Published:** 2015-05-19

**Authors:** Francesco Padormo, Arian Beqiri, Joseph V. Hajnal, Shaihan J. Malik

**Affiliations:** ^1^ Department of Biomedical Engineering, Division of Imaging Sciences and Biomedical EngineeringKing's College London, King's Health Partners, St Thomas’ HospitalLondonUK; ^2^ Centre for the Developing Brain, Division of Imaging Sciences and Biomedical EngineeringKing's College London, King's Health Partners, St Thomas’ HospitalLondonUK

**Keywords:** ultrahigh‐field MRI, parallel transmission, RF shimming, SAR, *B*_1_ mapping

## Abstract

The development of MRI systems operating at or above 7 T has provided researchers with a new window into the human body, yielding improved imaging speed, resolution and signal‐to‐noise ratio. In order to fully realise the potential of ultrahigh‐field MRI, a range of technical hurdles must be overcome. The non‐uniformity of the transmit field is one of such issues, as it leads to non‐uniform images with spatially varying contrast. Parallel transmission (i.e. the use of multiple independent transmission channels) provides previously unavailable degrees of freedom that allow full spatial and temporal control of the radiofrequency (RF) fields. This review discusses the many ways in which these degrees of freedom can be used, ranging from making more uniform transmit fields to the design of subject‐tailored RF pulses for both uniform excitation and spatial selection, and also the control of the specific absorption rate. © 2015 The Authors. *NMR in Biomedicine* published by John Wiley & Sons Ltd.

**Abbreviations used**
2D/3D: two/three dimensional
CoV: coefficient of variation
DSC: direct signal control
EPG: extended phase graph
EPI: echo planar imaging
FOV: field of view
FSE: fast spin echo
HW: hardware
LC: linear combination
LEx: local excitation
LLS: linear least squares
LTA: large tip angle
MaxMin: maximise the minimum
MLS: magnitude least squares
MP‐RAGE: magnetisation‐prepared rapid gradient echo
PatLoc: parallel imaging technique using local gradients
PNS: peripheral nerve stimulation
PSS: pseudo‐steady state
PTx: parallel transmission
RF: radiofrequency
ROI: region of interest
SAR: specific absorption rate
SLR: Shinnar Le‐Roux
SMS: simultaneous multi‐slice
SNR: signal‐to‐noise ratio
SPINS: spiral non‐selective
SR‐EPG: spatially resolved extended phase graph
SSE: spatially selective excitation
STA: small tip angle
TEM: transverse electromagnetic
TIAMO: time‐interleaved acquisition of modes
TSE: turbo spin echo
UHF: ultrahigh field
VERSE: variable rate selective excitation
VOP: virtual observation point.

## Introduction

Recent years have seen increased popularity of human MRI systems operating at ultrahigh magnetic field strength (*B*_0_ ≥ 7 T). However, operating at ultrahigh field (UHF) creates an additional set of technical challenges which need to be solved before it can be widely adopted. These problems originate from the interaction of the patient with the electromagnetic fields to which they are exposed during the course of an MRI examination. Although these interactions are present during examinations at lower field strengths, they are more severe for UHF MRI and therefore result in more significant image artefacts. The higher Larmor frequency (and consequently shorter electromagnetic radiation wavelength) results in wave interference effects becoming more pronounced 1, 2, 3, 4. This manifests itself as inhomogeneity in both the transmit and receive radiofrequency (RF) magnetic fields, *B*_1_^+^ and *B*_1_^–^. Although more spatially variable receive fields result in better parallel imaging performance 5, 6, non‐uniformities in the transmit field lead to spatially varying flip angles. This can result in spatially varying contrast and, in the worst cases, regions in which no excitation can be achieved at all. Furthermore, the precise pattern of inhomogeneity is subject dependent 7. A multitude of solutions have been proposed to address the problems associated with *B*_1_^+^ inhomogeneities, such as the use of adiabatic RF pulses 8, 9, dielectric pads 10, 11, 12 and dedicated coil designs 13, 14. However, the most flexible approach is the use of multiple transmission channels, known as parallel transmission (PTx), which is the subject of this review.

The concept of multiple transmitters was proposed by Hoult 15 and Ibrahim *et al*. 16 in 2000. The paper by Hoult investigated the fundamental limits of *B*_1_^+^ homogeneity achievable by the use of multiple coils to ‘shim’ the *B*_1_^+^ field in an analogous manner to *B*_0_ shimming. The first consideration of the use of multiple channels in a realistic loaded coil was performed by Ibrahim in a finite‐difference time‐domain numerical simulation study, in which improved *B*_1_^+^ field homogeneity was obtained with a birdcage coil by driving each rung with a different phase.

Interest in PTx increased greatly after the demonstration of RF pulse acceleration by Katscher *et al*. 17 and Zhu 18. It was these papers that realised that PTx could provide full spatial and temporal control of the RF field, an idea which the research community has latched on to with great enthusiasm. PTx has now transitioned from a purely research topic into clinical practice. Two channel transmitters are installed as standard in the latest clinical 3T systems from the major vendors, and many new 7T scanners are now equipped with multiple transmit channels.

This review explains the fundamentals and latest developments of PTx in its many different forms. This is achieved by classification of the methods based on the different time frames at which differences between channels are exploited. We begin with ’static PTx’, where the transmit settings are optimised once at the beginning of the experiment and then remain fixed for the rest of the scan. This is followed by ’dynamic PTx’, where differences between channels are exploited at the shortest time frames allowed by the system spectrometer. The intermediate area of ‘multi‐pulse PTx’ is then examined, followed by a discussion of further topics relevant to PTx.

## Fundamental Concepts

PTx systems differ from standard scanners by their RF system architecture. The key component of a PTx system is the transmit coil array, which must consist of several elements designed to produce spatially distinct RF field patterns. Each is driven by its own RF front end, consisting of multiple components. Although many different specific RF front ends have been proposed in the literature 19, 20, 21, 22, 23, for full PTx, all channels must be independently driven with full control over amplitude and phase modulation with microsecond temporal resolution. Each channel‐specific waveform requires a separate RF amplifier in order to deliver the required power to each coil array element.

When driven with an RF pulse, the *i*th transmit element produces RF magnetic and electric fields, denoted by ***B***_1,*i*_(**r**, *t*) and ***E***_*i*_(**r**, *t*), respectively. The NMR‐active component of ***B***_1,*i*_(**r**, *t*) is referred to as *B*_1,*i*_^+^(**r**,*t*) = ½[*B*_1,*i*_^x^(**r**,*t*) + *jB*_1,*i*_^y^(**r**,*t*)] 24, 25, where the *x* and *y* directions are perpendicular to the static magnetic field and *j* = √–1.

The different forms of PTx can be understood by further examining how they impact on the spatiotemporal nature of the transmit field. According to the principle of superposition, the net *B*_1_^+^ produced inside the subject is the sum of the fields produced by each element, as given by Equation (1a), where *N*_T_ is the number of transmit elements. However, the spatial and temporal components of *B*_1,*i*_^+^ can be separated, as shown in Equation (1b). Here, *S_i_*(**r**) is the spatial ‘footprint’ of a transmit element, often referred to as the transmit sensitivity, and *p_i_*(*t*) is the RF pulse played through the *i*th transmitter. This equation describes ‘dynamic PTx’, in which each coil element transmits its own channel‐specific waveform. A further simplification is shown in Equation (1c), where the same RF pulse waveform, *p*(*t*), is transmitted on each channel, scaled by a channel‐specific complex weight, *w_i_*. This equation describes ‘static PTx’. The final form of PTx described by this paper is ‘multi‐pulse PTx’, in which the channel‐specific weights or waveforms can change throughout an MR sequence.

(1a) $$B_{1}^{+}\left(r,t\right)=\sum_{i=1}^{N_{T}}B_{1,i}^{+}\left(r,t\right)$$

(1b) $$B_{1}^{+}\left(r,t\right)=\sum_{i=1}^{N_{T}}p_{i}\left(t\right)S_{i}\left(r\right)$$

(1c) $$B_{1}^{+}\left(r,t\right)=p\left(t\right)\sum_{i=1}^{N_{T}}w_{i}S_{i}\left(r\right)$$

All PTx methods rely on some degree of prior knowledge of *S_i_*(**r**) of each channel. This is achieved by ‘*B*_1_^+^ mapping’, which is discussed later in this article. Full knowledge of *S_i_*(**r**) constitutes the measurement of its amplitude and its phase relative to every other element at every location in space. It should be noted that there are many ways to define the units of *S*(**r**), *w_i_* and *p*(*t*) in a dimensionally consistent manner; the specific selection by a user will probably depend on the specifics of the PTx system being used. Unless stated otherwise, the figures in this paper consider sensitivity maps as dimensionless.

The electric fields generated by each element also play an important role in PTx experiments, as it is the electric field which is responsible for heating, with the specific absorption rate (SAR) used as a surrogate metric. Regulatory agencies place limits on temperature increases and on local and whole‐body SAR 26. SAR is a particular concern with PTx MRI because the total electric field (which is the result of a linear superposition of fields from each transmit channel) becomes spatially and temporally variable, potentially making ‘hot spots’ in unexpected locations. Many of the methods described in this review attempt to explicitly control SAR, often by using electric field information taken from numerical models. SAR is discussed in more detail later in the review.

Once the desired level of transmit and electric field information has been collected or inferred, it can be used to design the inputs to each of the transmit channels. Much PTx research has focused on the design of channel inputs with two separate goals in mind: (i) to overcome the effects of *B*_1_^+^ inhomogeneity; and (ii) to achieve local excitation (LEx).

*B*_1_^+^ inhomogeneity compensation can be achieved by all three forms of PTx. In this review, we adopt the following terminology for clarity: *B*_1_^+^ shimming refers to the use of static PTx to produce a spatially uniform overall *B*_1_^+^ field; flip angle shimming refers to the use of dynamic PTx to produce a spatially uniform flip angle; and signal shimming refers to the use of multi‐pulse PTx to ensure that each tissue type produces a spatially uniform signal in any measured image.

However, it is important to note that, whatever the desired goal (i.e. compensation for *B*_1_^+^ inhomogeneity, achievement of LEx or something more elaborate), there are generic algorithms with which the channel inputs can be determined. These different methods are described in the following sections.

## Static PTx

The most basic form of parallel transmission is static PTx, whose goal is to create the optimal conditions in a region of interest (ROI) by adjusting the complex weights (*w_i_*) with which the individual channels are driven, defined by Equation (1c).

What constitutes ‘optimal’ depends on the specific application, but the majority of static PTx demonstrations have focused on *B*_1_^+^ shimming. Initial methods specified the objective of uniform *B*_1_^+^ across the slice or volume being imaged 15, 16, 27, 28, 29. Alternative pragmatic approaches were simultaneously being explored experimentally. The first physical implementation of *B*_1_^+^ shimming demonstrated that the *B*_1_^+^ field could be optimised in specific voxels using a two‐port birdcage coil at 3 T 30. It was also shown that manual *B*_1_^+^ shimming using operator intervention yielded more uniform images at UHF 31, 32, 33. However, it was soon realised that demanding uniformity across the entire imaged slice can be overly restrictive. One way of improving performance is to demand uniformity only over a smaller ROI – this particularly makes sense when imaging structures that are smaller than the field of view (FOV) and was originally explored in the context of 7T prostate imaging 34, 35. As phase typically varies slowly in space, simply aligning the average phase of each channel within the ROI often leads to a good solution 35, as all channels are constructively interfering within the target region. This method has the advantage of not requiring the measurement of full *B*_1_^+^ information. The second realisation to aid *B*_1_^+^ shimming was that the appearance of an image often critically depends on the magnitude of the transmit field, and that its spatial phase distribution is often unimportant. Hence, *B*_1_^+^ shimming algorithms could relax their constraints, enabling solutions with a more homogeneous magnitude and inhomogeneous phase to be found 29, 36, 37, 38.

Other static PTx methods have been designed with specific applications in mind. For example, adiabatic pulses are only effective when the *B*_1_^+^ amplitude is above the adiabatic threshold. Therefore, methods have been designed to maximise the minimum (MaxMin) *B*_1_^+^ without constraining uniformity, so that the adiabatic condition is met across the object 39, 40. Another static PTx method obtains the weights which produce the largest overall *B*_1_^+^ amplitude per unit power deposited in the patient 41.

The use of static PTx for LEx has also been explored 42, 43, 44, 45. The utilised cost functions typically attempt to maximise the ratio of the *B*_1_^+^ field in a desired ROI to the *B*_1_^+^ field outside the ROI. These methods have not yet been widely adopted, as they require a large number of transmit channels to achieve the required localisation in order to perform reduced FOV imaging; instead, LEx usually requires the design of full RF waveforms via dynamic PTx, as discussed later.

The majority of static PTx methods determine optimum weights by solving a numerical optimisation problem, typically by iteratively minimising a cost function. Many different cost functions have been proposed, with most consisting of error terms to constrain the spatial *B*_1_^+^ distribution (*Φ*_target_), limit SAR (*Φ*_SAR_) and ensure the results are within hardware limits (*Φ*_HW_).

Static PTx optimisation problems are posed in one of three ways. The first is the regularised optimisation approach, in which the different penalised terms are added together, weighted by the regularisation factors *λ* and *μ*, as given in Equation (2a). The cost function is typically solved for multiple values of *λ* and *μ*; these form a family of solutions with differing trade‐offs between cost terms, often visualised using ‘L‐curves’ showing the size of the component error terms. The solution that offers the best compromise is selected. The second related approach is the constrained optimisation framework (Equation (2b)). This enables the optimum of the *B*_1_^+^ constraint term to be found for given SAR and hardware limits.

(2a) $$\text{minimise}\;\Phi_{\text{tot}}=\Phi_{\text{target}}+\lambda \Phi_{SAR}+\mu \Phi_{HW}$$

(2b) $$\begin{array}{c} \text{minimise}\;\Phi_{\text{target}}\\ \text{subject}\;to\;\Phi_{SAR}<SAR_{\text{max}}\\ \Phi_{HW}<HW_{\text{max}} \end{array}$$

A selection of the different *Φ*_target_ used in different static PTx methods is given in Table 1. The sensitivity matrix **S** is constructed from the *B*_1_^+^ field information of all *N*_T_ channels in a user‐defined ROI of *N*_ROI_ voxels. The information in the ROI from the *i*th transmitter is reshaped into an *N*_ROI_ × 1 column vector **s** *_i_*, all of which are horizontally concatenated, so that $S=\left[s_{1},s_{2},\ldots ,s_{N_{T}}\right]$. The *N*_T_ × 1 vector **w** contains the complex weights to each channel, and *N*_ROI_ × 1 vector **b** contains the desired *B*_1_^+^ field distribution.

**Table 1:** Example cost function terms *Φ*_target_ used to constrain *B*_1_^+^ in static parallel transmission (PTx) optimisation problems

Field constraints	Cost function term	References
Linear least squares (LLS)	${\left\|Sw-b\right\|}_{2}^{2}$	15, 16, 27, 28, 29
Magnitude least squares (MLS)	${\left\|\left|Sw\right|-b\right\|}_{2}^{2}$	36, 37, 38
Coefficient of variation (CoV)	$\frac{std\left(\left|Sw\right|\right)}{\text{mean}\left(\left|Sw\right|\right)}$	29, 34
Maximise the minimum (MaxMin)	max(min(|**Sw**|))	39, 40

Details of the SAR constraints can be found in the SAR section later in this review. For the sake of brevity, the additional hardware constraints are not described further, except to note that the most common additional constraint is for peak instantaneous power, which is related to max{|**w**|}; detailed explanations are given in refs. 46, 47, 48.

The third approach to obtain weights is to calculate them algebraically. These approaches do not require the use of optimisation algorithms as they can be found by simple arithmetic 35 or by performing matrix eigendecompositions 41.

**Figure 1:**
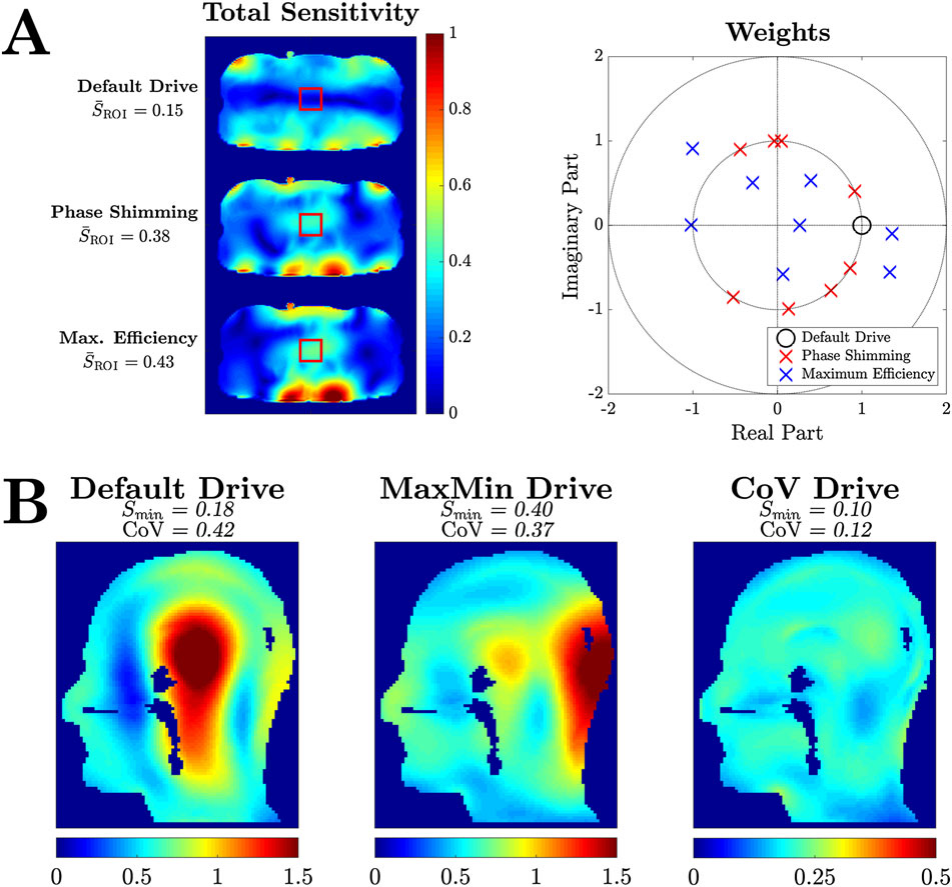
Illustrative example of *B*_1_^+^ shimming. (A) Net transmit sensitivities produced by a 7T, eight‐channel dipole array when transmitting with default drives (top left), phase shimming (centre left) and maximum efficiency (bottom left). The prostate is indicated by the red box. The average sensitivities in the regions of interest (ROIs) are given by ${\bar{S}}_{ROI}$. The weights obtained for each method are given on the right. (B) Net transmit sensitivities produced by a 7T, 12‐channel transverse electromagnetic (TEM) array with default drives (left), static parallel transmission (PTx) weights which maximise the minimum *B*_1_^+^ (centre), and applying weights which minimise the coefficient of variation (right). The minimum sensitivity in each slice is given by *S*_min_. (Data courtesy of Dr Alessandro Sbrizzi, Dr Alexander Raaijmakers and Dr Hans Hoogduin, UMC Utrecht, the Netherlands).

Illustrative examples of several static PTx strategies are shown in Fig. 1. Figure 1A considers prostate imaging at 7 T using an eight‐channel dipole array 49. Transmitting with the default weights of unit amplitude and zero phase (relative to an arbitrary reference) on each channel produces an overall *B*_1_^+^ field which is high at the periphery and low in the ROI. Phase shimming results in a larger *B*_1_^+^ field in the ROI, but the greatest field focusing is achieved using the maximum efficiency method. It should be noted that all solutions are scaled to have the same power (where power = **w** ^H^ **w**). Examining the weights themselves, the phase shimming algorithm is constrained to produce weights with the same amplitude for all channels. The maximum efficiency approach can reweight channels appropriately to produce a more efficient result. However, it should be noted that phase shimming does not require full *B*_1_^+^ field information, which can be difficult to acquire at 7 T.

Figure 1B demonstrates two static PTx methods for a sagittal brain slice using simulated data from a 12‐channel transverse electromagnetic (TEM) head coil. Again, it should be noted that all solutions are scaled to have the same power. The default weights produce a field with a very large dynamic range. The MaxMin algorithm produces a *B*_1_^+^ field which maximises the lowest realised field in the slice, providing improved performance for adiabatic pulses. However, the overall non‐uniformity remains, making these weights inappropriate for imaging using non‐adiabatic pulses. Using the coefficient of variation (CoV) metric produces a much more uniform *B*_1_^+^ appropriate for imaging, but with a much lower mean amplitude as the solution is very inefficient in terms of power.

The efficacy of static PTx has been widely demonstrated, particularly for two‐channel birdcage systems at 3 T, where *B*_1_^+^ shimming has resulted in improved and more reliable imaging in many different clinical imaging scenarios 50, 51, 52, 53, 54, 55, 56, 57, including imaging near metal implants 58. Furthermore, it has been shown that increasing the number of channels from two to eight improves the performance of 3T *in vivo* body imaging 59, and further studies have shown further improvements with up to 32 channels 60, 61. *B*_1_^+^ shimming has also been applied *in vivo* at UHF. Much work has focussed on the brain, with multiple imaging demonstrations at both 7 T 62, 63, 64, 65, 66 and 9.4 T 19, 33, 67, 68, 69, in addition to spectroscopy 70, 71, 72, 73. 7T body imaging has increased in popularity, with *B*_1_^+^ shimming being applied to cardiac 74, 75, 76, 77, 78, 79, musculoskeletal 41, 80, 81, 82, 83, prostate 35, 49, 84, 85, 86, liver 87 and kidney 88, 89, 90 imaging.

## Dynamic PTx

Static PTx is fundamentally limited to using the principle of superposition to achieve the goals of the pulse designer. Although it provides considerable control over *B*_1_^+^, for many imaging scenarios, the ability to achieve the desired *B*_1_^+^ across large FOVs at UHF is limited by the degrees of freedom provided by constructively and destructively interfering a finite number of transmit sensitivities 29, 60, 61. However, additional flexibility can be gained by recognizing that what is actually desired is a ‘flip angle’ distribution, which depends on the overall rotation of the magnetisation and not just the instantaneous *B*_1_^+^. Dynamic PTx modulates the *B*_1_^+^ field distribution over the shortest timescales, with the aim of directly controlling the rotation of magnetization, and hence the overall flip angle, at multiple spatial locations simultaneously. In this framework, the capabilities of PTx can extend far beyond that which is achievable with static PTx alone.

The behaviour of magnetisation is described by the Bloch equation. However, its non‐linear behaviour in the transverse magnetisation when rotations are large is difficult to incorporate into pulse design algorithms, and so the small tip angle (STA) approximation is often made 91. This simplifies the pulse design problem and introduces the concept of ‘transmit *k*‐space’ (**k**(*t*), as defined by Equation (3)) to account for the effect of magnetic field gradients applied during the RF pulse.

(3) $$k\left(t\right)=-\gamma \int_{t}^{T}G\left(t^{\prime }\right)\;dt^{\prime }$$

Here *T* is the duration of the pulse and **G**(*t*) is the applied field gradient on all three axes. The key difference between transmit *k*‐space and the more often used quantity for image encoding is the limits on the integral; in the transmit case, the integration runs from ‘now into the future’, whereas, for image encoding, the limits run from ‘the end of the excitation until now’. This can be understood by considering that, as the RF pulse is played out, new transverse magnetisation is being created, which is then subject to all future applied gradients. The STA approximation is used for the majority of current PTx pulse design algorithms 17, 18, 92, with the spatial domain approach 92 widely adopted as it is sufficiently flexible to incorporate *B*_0_ inhomogeneity, arbitrary transmit *k*‐space trajectories and spatial error weightings. As with static PTx, the pulses are obtained by minimising a cost function, posed in the same manner as Equation (2a) or (2b). An example using regularised optimisation with a linear least‐squares (LLS) error term and total RF power constraint is given by Equation (4). Here, **p** is a vector containing the RF pulses of all transmit channels, **m** is the desired transverse magnetisation vector and **A** is the system matrix, which contains all information about the excitation *k*‐space trajectory and the transmit field patterns. The terms in Equation (4) are closely related to those in cost functions used for static PTx. The vector **p** can be considered to contain time‐variable weights for the *N*_P_ intervals in the RF pulse; Equation (4) reduces to the static PTx optimisation for the case when *N*_P_ = 1.

(4) $$\text{minimise}\;\Phi_{\text{tot}}=\left\|Ap\right.-{\left.m\right\|}_{2}^{2}+\left.\lambda \right\|{\left.p\right\|}_{2}^{2}$$

This linear problem can be solved using a number of methods, such as conjugate gradients 93, and calculations can be accelerated by taking advantage of non‐uniform fast Fourier transforms 94. As with static PTx, many alternative cost functions have been proposed. If a spatially varying magnetisation phase is tolerable, the magnitude least‐squares (MLS) method can again be applied 37, 38. Optimisations that account for SAR 18, 46, 47, 95, 96, 97, 98, hardware 46, 48 or even thermal effects 99 can also be formulated in this framework, and these can be solved using regularised or constrained optimisations 46, 47.

Given the above possibilities in formulating the cost function, the remaining issue becomes the choice of *k*‐space trajectory. There are broadly two classes of trajectory – those used for flip angle shimming and those used for LEx. These are discussed in the next two sections.

### Dynamic PTx for flip angle shimming

**Figure 2:**
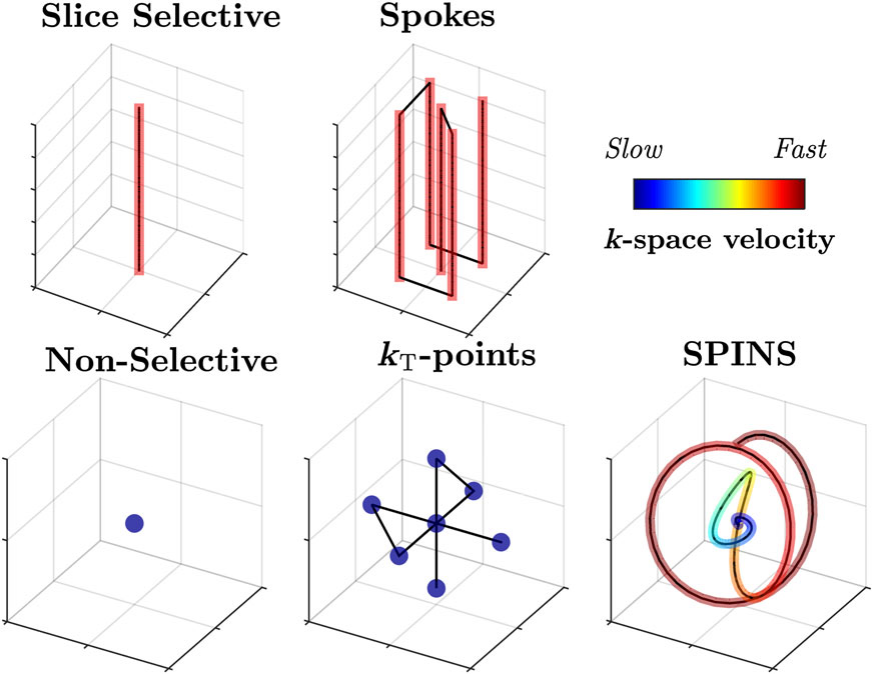
*k*‐space trajectories for flip angle shimming. Black lines indicate the path through *k*‐space, and shaded regions indicate where radiofrequency (RF) transmission occurs, with the colour of the shading indicating the *k*‐space velocity at that point. The top row shows trajectories which are spatially selective in a single direction and, consequently, much higher spatial frequencies are visited in that dimension.

**Figure 3:**
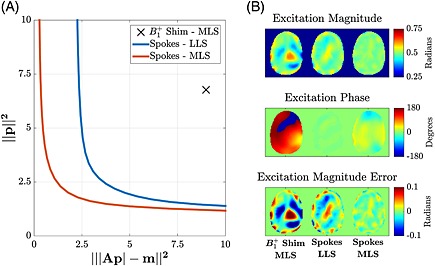
(A) L‐curve showing the trade‐off between power and homogeneity for a five‐spoke flip angle shimming pulse solved using linear least squares (LLS) and magnitude least squares (MLS). The best MLS *B*_1_^+^ shimming result is also shown. (B) Excitation magnitudes (top), excitation phases (centre) and the magnitude error (bottom) with respect to the target excitation of 0.5. The displayed spokes solutions were chosen to have the same power as the best MLS *B*_1_^+^ shimming solution. (Data courtesy of Dr Alessandro Sbrizzi and Dr Hans Hoogduin, UMC Utrecht, the Netherlands.)

PTx pulse design has been employed in cases in which *B*_1_^+^ shimming cannot achieve a sufficiently uniform flip angle across an ROI. A variety of different *k*‐space trajectories have been proposed, falling broadly into the two categories shown in Fig. 2; those that require slice or slab selection (top row), and those which can be non‐selective (bottom row). The selective trajectories are formed of individual ‘spokes’ which each provide slice selectivity – a single spoke is equivalent to a single slice‐selective pulse. Additional in‐plane spatial modulation is achieved by employing multiple spokes that are offset in k‐space; typically, these offsets correspond to low spatial frequency modulations, reflecting the spatial length scale of the *B*_1_^+^ inhomogeneity 38, 100, 101, 102, 103. The same RF waveform is used along each spoke in order to retain selectivity; consequently, the only quantities to be optimised are complex weightings of each subpulse for each channel, making these optimisation problems inherently two‐dimensional (2D) in nature. An example spoke RF pulse optimisation is illustrated in Fig. 3, which compares MLS *B*_1_^+^ shimming with a five‐spoke RF pulse, solved using both LLS and MLS approaches discussed earlier and additionally employing the multi‐shift algorithm 104. The L‐curve in Fig. 3 shows the trade‐off between flip angle accuracy (horizontal axis) and power (vertical axis) when solving the optimisation problems with different regularisation parameters. The MLS *B*_1_^+^ shimming result is only able to produce a moderately uniform field. The LLS spokes method produces an excitation with a more uniform magnitude, but is constrained to produce uniform phase. The MLS spokes method produces the most uniform excitation by relaxing the phase constraint.

If spatial selectivity is not necessary, simple hard pulses are often employed (which correspond to a point at ***k*** = 0). Low‐frequency *k*‐space modulations can also be introduced in three dimensions; the *k*_T_‐points method is a direct generalisation of 2D spokes, with the trajectory ‘stopping’ at discrete locations in *k*‐space 105. Alternatively, the ‘spiral non‐selective’ (SPINS) method uses a continuously moving three‐dimensional (33D) spiral trajectory to cover a low‐frequency 3D *k*‐space at variable velocity 106.

**Figure 4:**
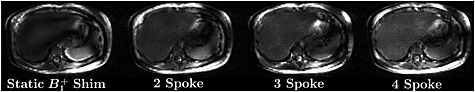
Liver imaging at 7 T with increasing numbers of spokes. Gradient echo images were each obtained within a single breath hold. In each case, homogeneity was optimised over the liver only and the images were processed to remove the receive field profiles. Homogeneity in the liver improves with an increasing number of spokes. (Images courtesy of Dr Xiaoping Wu, University of Minnesota, MN, USA, originally from Quant. Imaging Med. Surg. 2014; 4: 4–10, with permission.)

**Figure 5:**
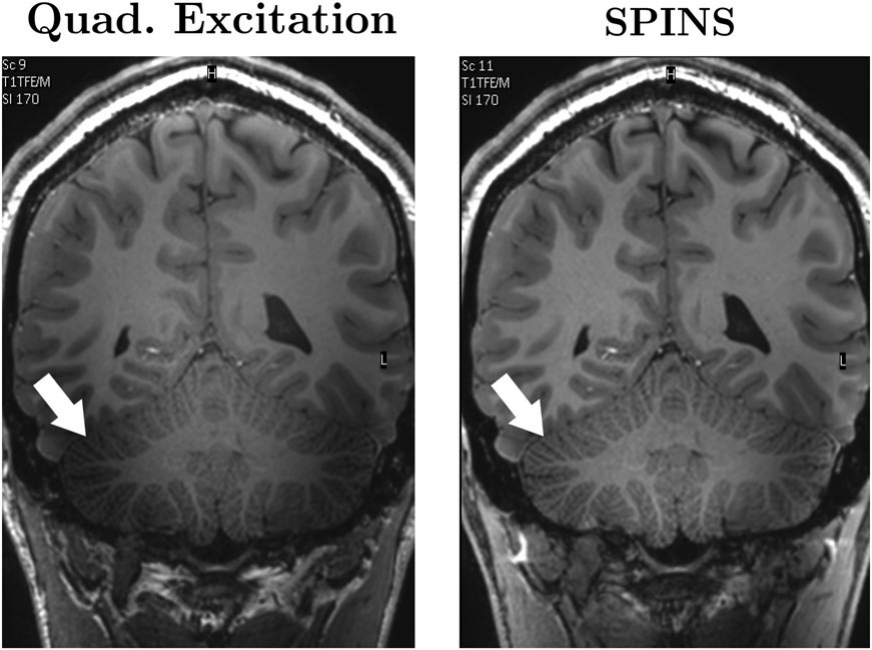
Matched coronal slices from *T*_1_‐weighted magnetisation‐prepared rapid gradient echo (MP‐RAGE) images acquired at 7 T using standard non‐selective and spiral non‐selective (SPINS) radiofrequency (RF) pulses with a two‐channel head transmit coil. Both were acquired at an isotropic resolution of 0.8 mm with a flip angle of 8°, shot interval of 3.5 s, inversion delay of 1.2 s, TR = 9 ms, TE = 2.9 ms, parallel imaging reduction factors of 1.3x2 (anterior‐posterior x right‐left) with a total imaging time of approximately 10 minutes in both cases. Image uniformity was seen to have improved throughout the head, particularly in the cerebellum, as shown here. (Images courtesy of Dr Hans Hoogduin, UMC Utrecht, the Netherlands.)

Flip angle shimming PTx pulses have been applied to both brain and body imaging. 2D spokes have been shown to improve 7T brain 66, cardiac 74 and liver 107 imaging. Figure 4 shows liver images obtained at 7 T using spokes pulses with increasing numbers of spokes – the achievable homogeneity increases at the expense of increasing pulse durations. 3D non‐selective pulses have also been shown to benefit high‐field brain imaging 108, 109. Figure 5 shows magnetisation‐prepared rapid gradient echo (MP‐RAGE) images acquired using SPINS excitation pulses with a two‐channel PTx system at 7 T. The relatively large amount of gradient encoding employed by SPINS pulses means that they can be effective with potentially a small number of transmit channels; at 3 T, good performance was demonstrated using only a single transmit channel 106.

### Spatially and spectrally selective PTx pulses

Another application of dynamic PTx pulse design is to reduce the duration of very long pulses, such as those used for LEx. Indeed, this is one of the primary applications originally envisaged for PTx 17, 18 because of the direct analogy with parallel imaging. LEx pulses are inherently lengthy, as their *k*‐space trajectories must visit high spatial frequencies in order to restrict the excited magnetisation to a small area. PTx allows the excitation *k*‐space to be undersampled, with multiple transmit channels used to avoid aliasing, as is the case for image encoding with parallel imaging. Acceleration of such pulses is important, not only in order to make them usable within rapid sequences, but also in order to reduce relaxation and off‐resonance effects.

**Figure 6:**
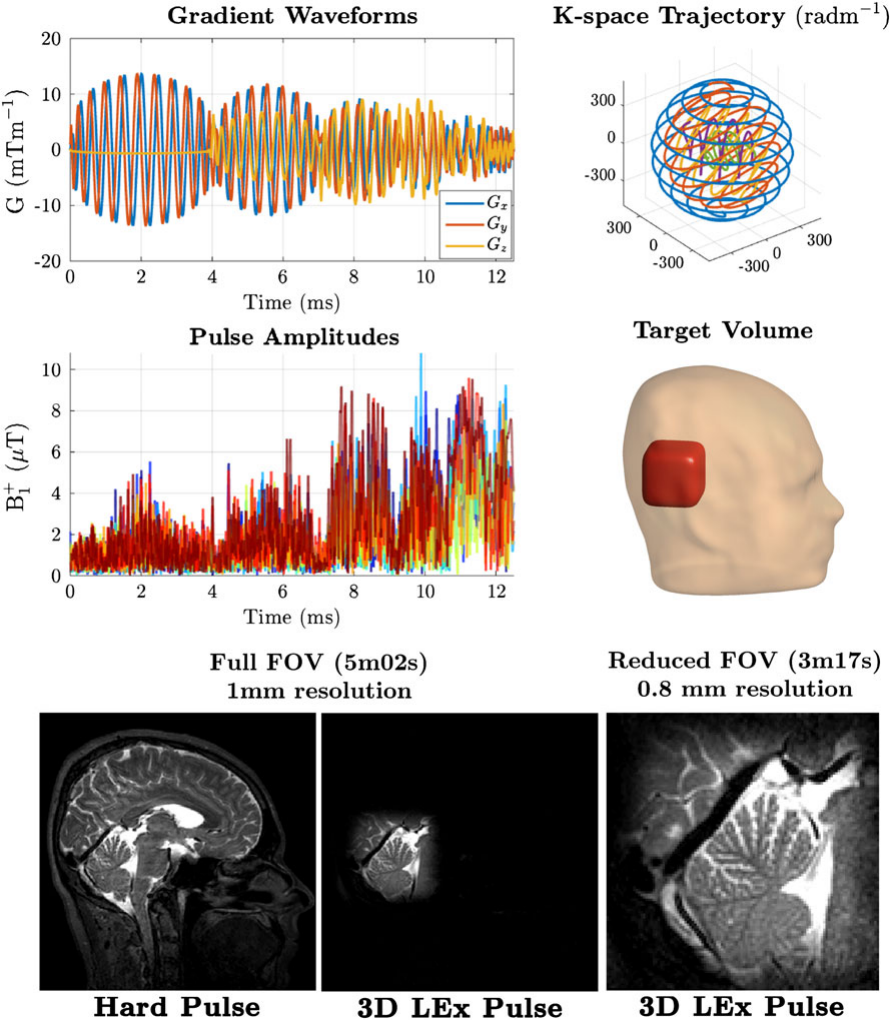
Three‐dimensional eight‐channel parallel transmission‐local excitation (PTx‐LEx) pulse design for three‐dimensional (3D) fast spin echo imaging at 3 T 116. The gradient waveforms (top left) result in a 3D shells excitation *k*‐space trajectory [top right, ref. 221] consisting of multiple nested shells that are coloured separately here for clarity. The overall pulse duration is 12.3 ms. The pulses (middle left, different colours indicate different channels) are designed to produce a 90° excitation in the target volume placed over the cerebellum (middle right). Full field of view (FOV) image using non‐selective hard pulse excitation (bottom left; isotropic resolution of 1 mm with parallel imaging reduction factors of 1.7x1.7 (anterior‐posterior x right‐left), full FOV image using designed LEx (bottom middle) and reduced FOV image using LEx (bottom right, isotropic resolution of 0.8 mm without parallel imaging).

PTx–LEx pulses have been an active area of development since the first demonstrations of the technique in post‐mortem animal models at 4.7 T 110 and in humans at 3 T 111. PTx–LEx used for the purpose of reduced FOV imaging has so far found limited applications, perhaps as reducing the number of measurements results in a loss of signal‐to‐noise ratio (SNR), and this approach directly competes with standard receive side parallel imaging. However, as with parallel imaging, performance could be expected to be improved with the increased SNR available with UHF MRI. PTx–LEx may prove to be particularly useful in situations in which avoidance of artefacts is key, for example by not exciting moving structures, as demonstrated in rodents 112 and humans at 7 T 113, 114. Another issue is that pulse durations, even after PTx acceleration, are typically still too long for use with rapid gradient echo or steady state free precession imaging sequences, but LEx may find a more natural use in other sequences, such as pulse‐acquire spectroscopy, where TR is sufficiently long to tolerate a long RF pulse, or 3D fast spin echo (FSE) imaging, where only the excitation pulse needs to achieve spatial selectivity alongside subsequent non‐selective refocusing pulses 115, 116, as demonstrated in Fig. 6. Clinical implementations of PTX–LEx have also been demonstrated at 3 T, focusing on abdominal diffusion‐weighted imaging applications where smaller FOVs can be used, reducing the length of the utilised echo planar imaging (EPI) readouts 117, 118, 119.

All RF pulses are spectrally selective to some degree, with bandwidth inversely related to duration; hence, typically, long LEx pulses tend to have narrow bandwidths. It is straightforward to add spectral selectivity to the Fourier STA approximation formalism as just another dimension whose associated ‘*k*‐space’ variable is time 120. This property has long been exploited to produce spectral–spatial‐selective pulses, such as binomial pulses often used for water or fat excitation. In fact, spokes pulses are identical in form to slice‐selective spectral–spatial pulses with the addition of in‐plane gradients. It has also been demonstrated that PTx can be used to achieve different excitation properties in water and fat, and to compensate for *B*_0_ effects 121, 122, within the same STA approximation design framework. With these methods, the additional degrees of freedom offered by PTx are being used to affect the spatial variation of the spectral response of the pulse by producing fields that are modulated in both space and time. If pulses are designed without accounting for frequency, they are in fact only controlling the on‐resonance response. The inclusion of *B*_0_ information in the design process is successful for single chemical species materials; however, materials with different chemical shifts, such as fat, will have uncontrolled excitation properties. This can be remedied by solving over a range of frequencies 123 in order to extend the bandwidth, or indeed by solving only for particular frequencies of interest, such as water and fat 124.

There are many possible additional extensions to the spatial domain view of pulse design. For example, PTx has been employed to overcome EPI signal drop out present as a result of through‐slice dephasing 125, 126, 127 by producing excitations that have the opposite phase variation through the slice and hence give refocused images at the echo time. Recent interest in the use of simultaneous multi‐slice (SMS) excitations 128 with UHF MRI for ultra‐high resolution diffusion and functional imaging 129 has also led to challenging pulse design problems as a result of the inherent high RF power and low bandwidth, in addition to *B*_1_^+^ inhomogeneity. Many methods incorporating SMS with PTx have been proposed and demonstrated at UHF 130, 131, 132, 133, 134, taking advantage of the distributed nature of PTx arrays to reduce peak RF power (a particular issue for SMS pulses), SAR and improve homogeneity. A recent study also proposed extending the spatial domain to include different ‘virtual slices’ that can be used to account for *B*_1_^+^ variation over the breathing cycle 135 – this approach extends the spatial domain to also include different respiratory phases, and improved robustness against respiration‐induced errors was demonstrated.

### *k*‐space trajectory optimisation

The design methods outlined so far give an optimal RF waveform for a given trajectory, but, as only the resulting effect on the magnetisation is of any real importance, it would make more sense to consider the trajectory itself as also subject to optimisation. Unfortunately, although the RF design problem can be cast straightforwardly as the inversion of a matrix problem, the trajectory enters into the design matrix itself, and therefore alternative methods are required to find both the optimal RF pulse and trajectory together.

A common approach is to create trajectories for classes of desired excitation pattern or for different RF coils that are manually or semi‐automatically optimised. For example, SPINS pulses 106 were designed initially for inhomogeneity correction at 3 T, where the desired response is typically a dome shape in 3D; a low‐frequency 3D spiral *k*‐space trajectory is therefore a natural choice. The *k*‐space locations for the rather simpler spokes 136 and k_T_‐points 105 trajectories are also often decided on the basis of similar arguments, for example using a ‘Fourier’ method that selects *k*‐space locations which correspond to the highest energies in the Fourier transform of the target pattern.

A more rigorous strategy is to parameterise the trajectory and to formulate the pulse design problem as an optimisation over both the RF samples and trajectory parameters iteratively. This approach has been explored for 2D 137, 138 and 3D 139 trajectories. Yip *et al*. 139 showed that this type of optimisation leads to intuitively reasonable results – for example, altering an EPI trajectory to increase the sampling density in regions in which the Fourier transform of the target excitation has higher energy. A drawback is that the additional level of optimisation results in increased computation times, particularly as the trajectory optimisation is a non‐linear problem. This issue is likely to be more significant for 3D problems, or when there are a large number of trajectory parameters, but simplifies when applied to spokes/*k*_T_‐points pulses. Zelinski *et al*. 140 showed that optimising by enforcing ‘sparsity’ of *k*‐space locations helps to reduce the number of required spokes and performs better than the simple Fourier approach. The choice of multiple locations simultaneously is a computationally complex problem; Ma *et al*. 141 proposed a fast greedy algorithm which chooses locations one at a time, which reduces these requirements considerably. The frequency selectivity of the pulse is also determined by the time ordering of the locations chosen, and so an updated greedy algorithm was proposed to also take this into account 142.

As well as excitation fidelity, trajectories can also be optimised to minimise the required RF power. For example, spiral trajectories designed for 2D excitation have employed lower slew rates towards the centre of *k*‐space 98, 143 in order to reduce instantaneous RF power, similar to applying variable rate selective excitation (VERSE) 144. An alternative solution is to numerically optimise a trajectory based on properties of the target 145, 146. A more comprehensive approach for constraining peak instantaneous power was proposed by Lee *et al*. 147 who modified a time‐optimal implementation of the VERSE algorithm 148 by transforming the constraint on peak RF power, so that it could be included as a gradient constraint. An issue with VERSE is that time dilating RF pulses changes their off‐resonance properties. Lee *et al*. 149 proposed an updated version of their method which iteratively alters the RF design after time dilation to counter this issue.

Of the methods discussed so far, some update *k*‐space on a per‐subject basis 137, 139, 147, 149, whereas others have a fixed trajectory (and hence gradient waveform) for all subjects 106, 143, 145, 146. Although the latter group is less flexible, these methods do avoid the performance of additional calculations whilst the subject is *in situ*. Another advantage of this latter approach is that gradient system imperfections can be calibrated in advance. PTx pulses with complex gradient waveforms are often more sensitive than standard pulses to gradient system errors 150, 151. In cases in which the waveforms do not change from subject to subject, these can be measured in advance with the true trajectory used for pulse design 106, 152; this is feasible because the required corrections have been reported to remain stable over long periods of time 106. Methods that adapt trajectories for each subject may need to incorporate gradient imperfections using models, for example by treating them as a linear time‐invariant system and employing an impulse response function 116, 153, 154. Waveform measurement using MRI and iterative predistortion 155 of waveforms require gradient measurements that can be performed quickly with the subject *in situ*; although image‐based methods are available (for example, ref. 156), this general approach is much more feasible if gradient probe measurements are available 157.

### Beyond the STA approximation

The STA approximation provides an elegant Fourier picture which is useful for discussion as well as for simplifying the design problem. However, the linear *k*‐space picture breaks down for large tip angles (LTAs) and, although some classes of *k*‐space trajectory can produce satisfactory results 158, more sophisticated design methods are required. Non‐PTx LTA pulse design can be performed using the Shinnar Le‐Roux (SLR) algorithm 159, 160, which was recently extended to multidimensional *k*‐space trajectories 161. However, other methods are required for LTA PTx design; many approaches have been proposed, and these typically incorporate *B*_1_^+^ field information. A simple extension to STA approximation pulse design is the ‘additive angle’ method 162, which uses STA approximation designed iteratively with a Bloch simulation, designing a new pulse at each stage to compensate for the errors of the previous one, and then summing all of these contributions at the end. This method can be improved upon by performing a perturbation analysis of the Bloch equations; the STA approximation is the first‐order term, but higher orders can be addressed iteratively to improve the design 163. These methods usually require multiple Bloch equation simulations to accurately model the magnetisation behaviour. The simplest case is that of ‘composite’ pulses, consisting of trains of a few non‐selective pulses; the solution to the Bloch equations here can be boiled down to a set of simple rotations, and these can be optimised numerically 164. More sophisticated pulses can be designed using optimal control methods, which solve dynamic optimisation problems with differential equations as constraints. These have a long history of use within MRI (for example, ref. 165), and have been used recently for PTx RF pulse design 108, 166, 167, 168, with much work carried out to reduce computation times and to find globally rather than locally optimal solutions.

Finally, the trend of parallelising the subsystems of MRI scanners has recently been extended to gradients. The parallel imaging technique using local gradients (PatLoc) 169 and O‐space 170 imaging offer the ability to image higher resolutions with lower peripheral nerve stimulation (PNS) by using non‐bijective gradients. PTx has been unified with these methods, but the Fourier domain picture does not apply because of the non‐linearity of the spatial gradients 171.

## Multi‐pulse PTx

So far, the methods discussed have either fixed the *B*_1_^+^ field pattern throughout an RF pulse or modulated it over very short timescales, during a single RF pulse. An intermediate timescale also exists: modulation between pulses in one single sequence, referred to here as multi‐pulse PTx. As discussed earlier, there is typically a trade‐off between achievable *B*_1_^+^ homogeneity and RF power/SAR. One use for multi‐pulse PTx is to apply this trade‐off flexibly within a sequence. Homann *et al*. 172 proposed switching static PTx weights mid‐scan between high SAR, good *B*_1_^+^ homogeneity settings when low‐frequency *k*‐space data are being acquired and low SAR, poor homogeneity settings when outer *k*‐space data are being obtained. This minimises the impact of poor *B*_1_^+^ homogeneity on image contrast which is dominated by the RF conditions when acquiring the centre of *k*‐space, whilst reducing the average SAR. Metzger *et al*. 90 used a similar principle for inversion‐prepared renal angiography at 7 T; low SAR weights are used for adiabatic inversion pulses as these can tolerate some *B*_1_^+^ inhomogeneity, but typically have high associated SAR, whereas high *B*_1_^+^ homogeneity weights are used for excitation pulses whose homogeneity directly affects image quality, but which have a lower overall impact on the sequence SAR. A related method is time‐interleaved acquisition of modes (TIAMO) 173, 174, which can be used to remove signal voids caused by regions of low or even zero *B*_1_^+^ employing a parallel imaging reconstruction to create a composite image of data acquired using interleaved static PTx drives with different spatial sensitivity patterns that shift low *B*_1_^+^ areas to different locations.

By their nature, MRI pulse sequences consist of many RF pulses, and the overall effect on the received signal depends on the interactions between these pulses and the spin system. All of the RF pulse design and shimming strategies discussed so far have treated each pulse in isolation; however, an alternative is to take a more integrated approach. One example is to design pairs of pulses together, which can be beneficial in situations in which pulse properties need to be ‘matched’, as demonstrated for spin echo excitation and refocusing pulse pairs 175 and for flip‐down/flip‐up pairs 176.

**Figure 7:**
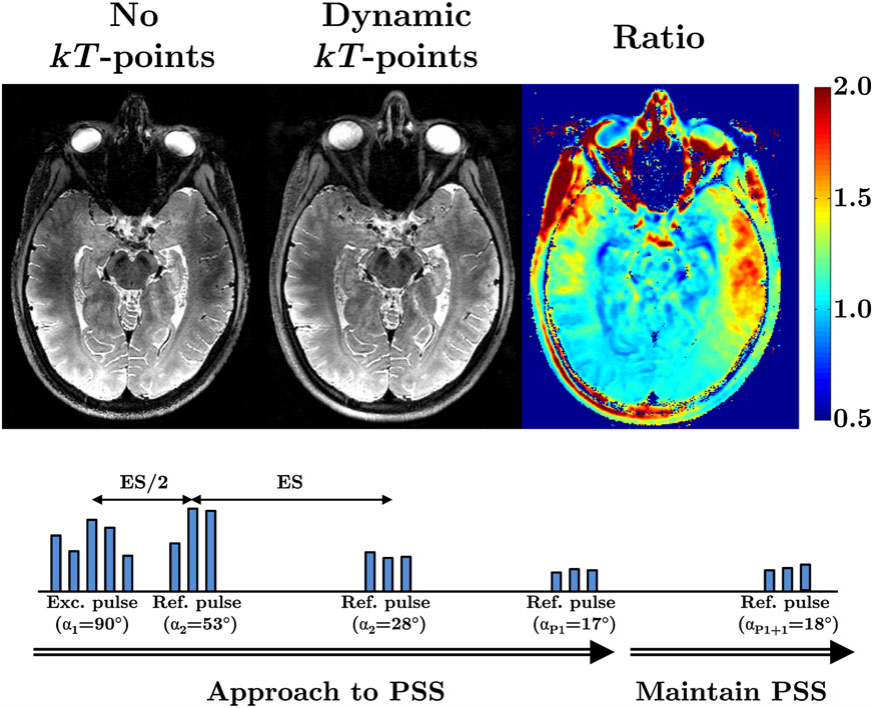
*T*_2_‐weighted three‐dimensional fast spin echo (FSE) imaging at 7 T using dynamically modulated *k*_T_‐points radiofrequency (RF) pulses for excitation and refocusing. The diagram (bottom) depicts the *k*_T_‐points RF pulses used, consisting of multiple hard pulses. The amplitudes and phases of these hard pulses are optimised so that, during each shot of the FSE sequence, the magnetization is brought to a pseudo‐steady state (PSS) with desired echo amplitude by the first P_1_ pulse (here P_1_ = 10), and then subsequently maintained in this state, despite the presence of strong *B*_1_^+^ non‐uniformity. The spatially resolved extended phase graph (SR‐EPG) framework is used to predict the echo amplitudes for all locations in space and at each TE, and these are optimized to be uniform 182. Dynamic modulation allows more uniform signals to be obtained, recovering reduced signals that are apparent in the temporal lobes (see increased signal apparent on the ratio image). (Images courtesy of Dr Florent Eggenschwiler, CIBM, Lausanne, Switzerland.)

In rapid imaging sequences, the magnetisation will reach a steady or pseudo‐steady state (PSS) after many RF pulses; it has been shown that dynamic modulation of the static PTx weights during FSE sequences 177 can lead to better image quality than static *B*_1_^+^ shimming alone. This approach, referred to here as direct signal control (DSC), is fundamentally different from those discussed so far. Although *B*_1_^+^ shimming typically seeks to control the *B*_1_^+^ field pattern, and flip angle shimming controls the transverse magnetisation at the end of an RF pulse, DSC may be thought of as ‘signal shimming’, where we seek to directly influence the signals that will be received during an imaging sequence consisting of multiple interacting RF pulses. One way of achieving this is by performing a non‐linear optimisation with respect to a signal model; for FSE sequences, this can be efficiently constructed from a spatially resolved extension to the well‐known extended phase graph (EPG) formalism 178. The method has been applied to 3D FSE imaging at 3 T 179, and related approaches which employ full PTx refocusing pulses have been demonstrated at 7 T 180, 181, 182, an example of which is illustrated in Fig. 7.

## SAR

Increased SAR is intrinsically a problem for UHF MRI and is an area in which PTx can have both positive and negative effects. As PTx results in spatiotemporal variations in electric (as well as magnetic) RF fields, it can change the expected locations of hot spots. If local SAR is not considered when performing PTx calculations, a significant risk of heating can result. Global (i.e. whole body averaged) SAR can be estimated using measurements of forward and reflected power 183, but the estimation of local SAR typically requires a knowledge of electric fields. These cannot currently be measured reliably by MRI (although it is an active research field 184, 185, 186, 187), and so this information is typically provided by numerically solving Maxwell's equations on a high‐resolution grid (typically 1–5 mm^3^) for digital body models. Once obtained, the *E*‐field data can be related to SAR using the Q‐matrix framework 188, which represents, in a matrix, the contribution to SAR from each possible combination of channels. For example, the instantaneous local SAR is given by Equation (5), where *σ*(**r**) is the tissue conductivity, *ρ*(**r**) is the tissue density and **Q**(**r**) is the Q‐matrix at location **r**. The global SAR matrix **Q**_global_ can be inferred by taking a weighted average of the local Q matrices 189.

(5) $$SAR_{\text{local}}\left(r\right)=\frac{\sigma \left(r\right)}{2\rho \left(r\right)}w^{H}Q\left(r\right)w$$

All parts of the body exposed to the RF fields must be considered when evaluating SAR, not just those in the imaging region. As the location of maximum local SAR can occur anywhere and is not known a priori, Equation (5) must be evaluated for every location in space to ensure that regulatory limits are met. This is time consuming because of the sheer number of matrices, often in the range of 10^6^–10^8^. This process can be significantly accelerated by taking advantage of the positive semi‐definite nature of the Q‐matrices to form a smaller subset (known as ‘virtual observation points’) of Q‐matrices, **Q**_VOP_, whose local SAR values dominate the calculation 190, 191. Compression factors of the order of 5000 have been demonstrated for human models with eight transmit channels 190 with the guarantee of no underestimation of max{SAR_local_} and a prescribed limit for overestimation, in this case 5%.

Much work has gone into understanding the exact properties of the required digital body models (for example, ref. 192). Many strategies have been proposed, including the production of patient‐specific whole‐body models based on *in situ* scans of a given subject 193, the creation of patient‐specific models by image registration 194 or the use of generic models with a suitably chosen conservative safety factor 195. Although the majority of proposed methods rely on some form of SAR model, others are also exploring the possibility of direct *in vivo* measurement by post‐processing *B*_1_^+^ maps, with results demonstrated at 3 T 196 and 7 T 185. Clearly, direct measurement of SAR would be ideal; however, these methods could only be practically used if the necessary data could be acquired quickly so as not to compromise the examination itself – this is made more challenging by the fact that areas of elevated SAR can occur far from the slice or volume that is being considered for imaging, and so SAR measurements will necessarily require large fields of view for many coil designs. Direct measurements of temperature increases are also being explored as a way of determining safe scanning using MR thermometry 187, 197.

Once SAR information has been obtained, it can be used to limit SAR within pulse sequences 90, 172, 173. For PTx pulse designs, this is achieved by incorporating SAR penalisation terms into the cost functions minimised to calculate the pulse waveforms (e.g. Equations [2] and (4)). Table 2 describes the SAR terms commonly used in static PTx optimisation problems. All involve a quadratic form of the weights and Q‐matrices. These terms can be easily generalised for dynamic PTx pulse calculations to limit local SAR 46, 47. This type of constraint is useful, given that it has been shown that maximum local SAR, in particular, can vary strongly when pulse design parameters are changed 198. It has also been proposed that pulse design can be used to directly constrain temperature rather than SAR, by combining SAR models with biophysical thermal models utilising the Pennes bioheat equation 99.

**Table 2:** Example cost function terms *Φ*_SAR_ used to constrain the specific absorption rate (SAR) in static parallel transmission (PTx) optimisation problems

Energy constraints	Cost function term	References
Total RF power (**I**, identity matrix)	**w** ^H^ **Iw**	92
Global SAR	**w** ^*H*^ **Q**_*global*_ **w**	18
Local SAR (index *i* runs over all spatial locations)	**w** ^H^ **Q**(**r**_*i*_)**w** ∀ *i*	46
VOP SAR (index *i* runs over all VOPs)	$w^{H}Q_{i}^{VOP}w\;\forall \;i$	191, 192

Rather than viewing SAR as a constraint, the reduction of SAR can be seen as the major target of any optimisation – Zhu 18 discussed this possibility in his early paper on PTx, and it has been shown that simultaneous reductions in local SAR and *B*_1_^+^ inhomogeneity can be achieved by performing *B*_1_^+^ shimming within localised ROIs for prostate imaging at 7 T 34 and for cardiac imaging at 3 T 199. A further application of PTx has been the control and reduction of SAR in the presence of implanted or interventional devices 200, 201, 202, 203. These methods use spatial control of RF electric fields, made possible by PTx, in order to minimise heating effects using simulated electric fields or *in situ* measurements of electrical coupling for optimisation. This is a promising application for PTx that has so far been of particular interest at lower field strengths.

Finally, it should also be noted that electric fields and SAR depend on the utilised RF coil. It has been shown that certain transmit arrays can be driven using a basis of circularly polarised modes 204, some of which produce very little *B*_1_^+^ yet significant electric fields. Although they produce very little *B*_1_^+^, these ‘dark modes’ can be used to cancel electric fields produced by the more *B*_1_^+^ efficient modes to reduce SAR hotspots 205. Taking this concept further, it is possible to design coil arrays with some dedicated ‘dark’ elements that primarily produce electric fields. An example of such a system is illustrated in Fig. 8 from ref. 206, where dipole antennas are employed in conjunction with loops. Although this design is unconventional, each element in this array is driven independently, and it may be used in exactly the same way as any other PTx array using any of the optimization methods outlined previously. It has been demonstrated that potentially large reductions in local SAR 206 can be achieved. Although these approaches are still in their infancy, previous theoretical studies into optimal current distributions suggest that there are significant benefits yet to be obtained 61, 207.

**Figure 8:**
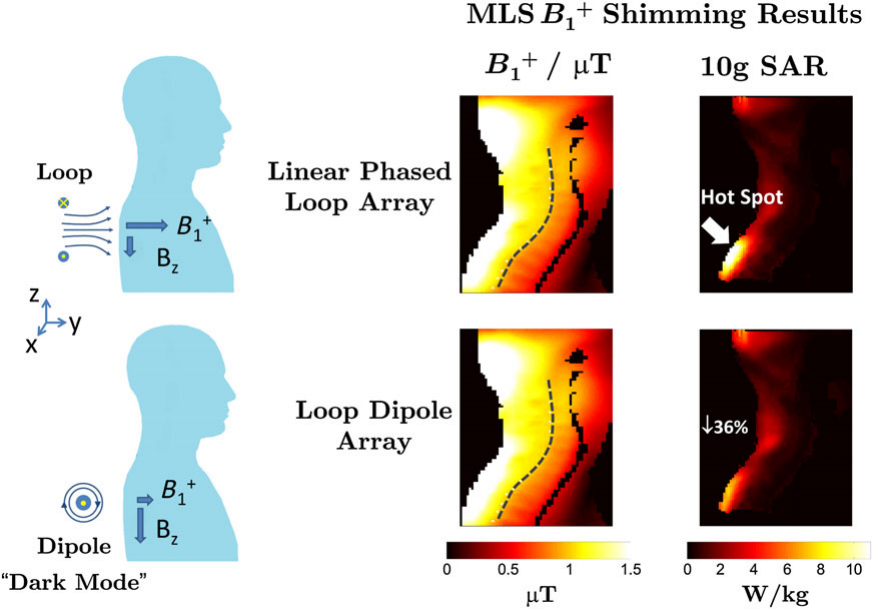
Specific absorption rate (SAR) reduction achieved by combining a loop and dipole array. The dipoles are orthogonal to the main magnetic field (*z*‐axis), and therefore produce radiofrequency (RF) magnetic fields primarily in the *z*‐axis – these produce very little contribution to the NMR and are hence referred to as ‘dark modes’. The electric fields produced by the dipoles can, however, be used to cancel those produced by the loops, thus reducing local SAR. In this example, magnitude least squares (MLS) *B*_1_^+^ shimming using an array of four loops and four dipoles was able to reduce peak 10 gram SAR by 36% when compared with a four‐loop linear phased array, whilst producing an almost identical *B*_1_^+^. (Images courtesy of Dr Yigitcan Eryaman, University of Minnesota, MN, USA. Data reproduced from Magn. Reson. Med, Epub 2014, doi: 10.1002/mrm.25246., with permission).

## *B*_1_^+^ Mapping

PTx methods inherently require some knowledge of the transmit field produced by each element of the transmit array. This information is typically acquired *in situ*, and many different strategies have been proposed. The most basic approach involves the utilisation of a sequence which measures the magnitude of the transmit field (for example, see refs. 208, 209, 210, 211), and repeating this for each transmitter. The relative phase of each transmitter is either obtained from the phase of the images acquired or, in some cases, from a dedicated acquisition 212. This approach is typically lengthy, as the majority of *B*_1_^+^ mapping methods (apart from recently proposed exceptions 211, 212, 213) are slow and, unlike receive field mapping, transmit channels must be mapped sequentially. Another approach is to acquire only a single magnitude transmit field map of all coils transmitting in a default configuration, supplemented by a series of low‐flip‐angle spoiled gradient echo images 214, 215 (whose signal is proportional to the *B*_1_^+^ field) from which relative transmitter information can be obtained. This technique is fast, as the data acquisition is very efficient with low SAR.

UHF *B*_1_^+^ mapping is more challenging than at lower field strengths, primarily because of the increased dynamic range of the transmit field; typically very large *B*_1_^+^ is produced adjacent to coil elements, with very low and often zero amplitudes produced further away within the FOV. All *B*_1_^+^ mapping methods have a limited range of flip angles over which they can acquire accurate measurements 216. In order to combat this, the use of linear combinations (LCs), constructed so as to reduce the dynamic range, has been proposed 217, 218. The choice of LC usually requires a trade‐off between reducing the dynamic range and the ability to invert the measurements 219. Recent work at 9.4 T has suggested that Fourier encoding is a good choice using an eight‐channel head coil 220, but work at 3 T has shown that the best choice of LC is coil array and load specific 219, and a suitable LC cannot always be found.

## Concluding Remarks

UHF MRI provides a powerful tool for investigation of the human body, for both clinical diagnosis and fundamental research. However, it brings a new set of challenges which need to be overcome. This review has focused on how the new degrees of freedom made possible by PTx can be used, focusing on different temporal scales over which RF fields can be modulated and different ways in which this interacts with MR image formation. We have touched upon some of the remaining technical challenges, such as accurate estimation of SAR and rapid *B*_1_^+^ mapping, but there are also additional RF engineering issues, such as optimal design of RF coils and amplifiers, that are beyond the scope of this review. Many of the methods discussed here have been proposed in methodological studies, but as PTx technology matures and becomes more widespread for UHF MRI, it is hoped that some will now prove their efficacy for routine *in vivo* research and clinical use.
